# Draft Genome Sequence of Rhodococcus qingshengii (Formerly *erythropolis*) TA37, a First-Generation Biocatalyst for Synthesis of Functionalized Acrylamides

**DOI:** 10.1128/MRA.01057-21

**Published:** 2021-12-16

**Authors:** Konstantin V. Lavrov, Andrey D. Novikov, Artem S. Kasianov, Stepan V. Toshchakov, Aleksei A. Korzhenkov, Alexander S. Yanenko

**Affiliations:** a NRC Kurchatov Institute, Moscow, Russia; b NRC Kurchatov Institute—GOSNIIGENETIKA, Kurchatov Genomic Center, Moscow, Russia; c Vavilov Institute of General Genetics, Moscow, Russia; Montana State University

## Abstract

We describe here the 7.0-Mb draft genome sequence of Rhodococcus qingshengii strain TA37, which was obtained from samples of nitrile-contaminated soil collected in the Saratov Region (Russian Federation). This genomic resource will support the further development of biocatalysts for the inexpensive and green production of acrylic monomers.

## ANNOUNCEMENT

Bacteria have accumulated a wealth of enzymes, which are able to perform many types of useful synthetic reactions. Several successfully commercialized biosynthetic processes have been initiated by isolating a bacterium with a specific enzyme activity, which was then identified at the genetic level and used in industry (1, 2). For nonconventional bacteria such as *Rhodococcus* species, genome sequencing now is indispensable both for linking the enzyme activity with its gene and for the further development of biocatalysts.

Here, we report the genomic data of *R. qingshengii* strain TA37 (formerly “R. erythropolis”; see below for species redefinition), which was used as a first biocatalyst for the amidase-catalyzed synthesis of functionalized acrylamides (3). The strain was isolated from acrylonitrile-polluted soil near Saratov, Russian Federation (51.540600, 46.008600). Minimal medium of the following composition was used for isolation of the strain: 1 g/liter KNO_3_, 0.6 g/liter KH_2_PO_4_, 1.4 g/liter Na_2_HPO_4_, 0.3 g/liter MgSO_4_, and 0.5% acrylonitrile (pH 7.2). Agar agar was added if needed (20 g/liter). Soil extracts were spread onto agar plates, and a group of colony-forming strains was selected after a 7-day incubation at 30°C. Among them, strain TA37 was selected due to its acylamidase activity, measured as described in reference 4.

For genomic DNA extraction, the strain was grown on nutrient broth (5 g/liter yeast extract, 5 g/liter tryptone, 2.5 g/liter NaCl [pH 7.2]) at 30°C for 2 days. DNA was extracted using the method described in reference 5, and a DNA fragment library was prepared using the KAPA HyperPlus kit according to the manufacturer’s recommendations. Sequencing was performed on the Illumina MiSeq system at NRC Kurchatov Institute (Moscow, Russia) using the MiSeq reagent kit v2, and 3,363,592 paired-end 251-bp reads were generated. During the read processing and genome assembly, default parameters were used for all software unless otherwise specified. Preassembly quality control of the reads was performed using FastQC v0.11.9 (https://www.bioinformatics.babraham.ac.uk/projects/fastqc/). The reads were quality trimmed (qtrim=r, trimq=18) and adapter sequences were removed using BBDuk. Overlapping reads were merged using BBMerge within BBMap (all BB programs were v38.86; https://sourceforge.net/projects/bbmap/). Then, the reads were assembled using SPAdes v3.14 (6) with the “careful” option, producing 136 contigs larger than 250 bp with an average 100-fold coverage. The total genome size was 6,980,438 bp, the GC content was 62.3%, and the *N*_50_ value was 410,730 bp.

During submission to NCBI GenBank, the genome was compared with the genomes of type strains that are already in GenBank using the average nucleotide identity test, as described in reference 7. It was found that the genome has the highest identity to the genome of *R. qingshengii* (BioProject accession no. PRJDB9718), with 98.773% identity and 87.2% genome coverage. Thus, strain TA37 was renamed from R. erythropolis to R. qingshengii.

Automatic annotation was performed using the NCBI Prokaryotic Genome Annotation Pipeline (PGAP) v5.2, generating a total of 6,643 genes, 6,572 potential protein-coding genes, 71 genes coding for RNAs, including 54 tRNA genes, 38 rRNA genes, 3 noncoding RNAs (ncRNAs), 84 pseudogenes, and 29 amidases. Due to the large number of putative amidase genes (only two of which were previously characterized [4, 8]), this genomic resource will support further development of biocatalysts for the inexpensive and green production of acrylic monomers.

### Data availability.

The draft genome sequence is available at NCBI GenBank under accession no. JAHREK000000000 and BioProject accession no. PRJNA740177, and the raw sequencing reads are available under SRA accession no. SRX11204876. The version described in this article is the first version.

## References

[1] Leonova TE, Astaurova OB, Ryabchenko LE, Yanenko AS. 2000. Nitrile hydratase of *Rhodococcus*: optimization of synthesis in cells and industrial applications for acrylamide production. Appl Biochem Biotechnol 88:231–241. doi:10.1385/ABAB:88:1-3:231.

[2] Kobayashi M, Shimizu S. 1998. Metalloenzyme nitrile hydratase: structure, regulation, and application to biotechnology. Nat Biotechnol 16:733–736. doi:10.1038/nbt0898-733. 9702770

[3] Lavrov KV, Larikova GA, Yanenko AS. 2013. Novel biocatalytic process of N-substituted acrylamide synthesis. Appl Biochem Microbiol 49:702–705. doi:10.1134/S0003683813080048.

[4] Lavrov KV, Zalunin IA, Kotlova EK, Yanenko AS. 2010. A new acylamidase from *Rhodococcus erythropolis* TA37 can hydrolyze N-substituted amides. Biochemistry (Mosc) 75:1006–1013. doi:10.1134/s0006297910080080. 21073421

[5] Sidoruk KV, Levitin EI, Sviridov BV, Piksasova OV, Shustikova TE. 2020. Extraction of DNA from a wide range of objects by treatment with ammonium salts. Biotekhnologiia (Moskva) 36:98–106. doi:10.21519/0234-2758-2020-36-6-98-106.

[6] Bankevich A, Nurk S, Antipov D, Gurevich AA, Dvorkin M, Kulikov AS, Lesin VM, Nikolenko SI, Pham S, Prjibelski AD, Pyshkin AV, Sirotkin AV, Vyahhi N, Tesler G, Alekseyev MA, Pevzner PA. 2012. SPAdes: a new genome assembly algorithm and its applications to single-cell sequencing. J Comput Biol 19:455–477. doi:10.1089/cmb.2012.0021. 22506599 PMC3342519

[7] Ciufo S, Kannan S, Sharma S, Badretdin A, Clark K, Turner S, Brover S, Schoch CL, Kimchi A, DiCuccio M. 2018. Using average nucleotide identity to improve taxonomic assignments in prokaryotic genomes at the NCBI. Int J Syst Evol Microbiol 68:2386–2392. doi:10.1099/ijsem.0.002809. 29792589 PMC6978984

[8] Lavrov KV, Karpova IY, Epremyan AS, Yanenko AS. 2014. Cloning and analysis of a new aliphatic amidase gene from *Rhodococcus erythropolis* TA37. Russ J Genet 50:1009–1016. doi:10.1134/S1022795414100056. 25720247

